# Exploring the barriers to implement industrial symbiosis in the apparel manufacturing industry: Implications for sustainable development

**DOI:** 10.1016/j.heliyon.2024.e34156

**Published:** 2024-07-05

**Authors:** Mosaddeque Hossain, Ridwan Al Aziz, Chitra Lekha Karmaker, Binoy Debnath, A.B. M. Mainul Bari, Abu Reza Md Towfiqul Islam

**Affiliations:** a Department of Industrial and Production Engineering, Bangladesh University of Engineering and Technology, Dhaka, 1000, Bangladesh; b Department of Disaster Management, Begum Rokeya University, Rangpur, 5404, Bangladesh

**Keywords:** Industrial symbiosis, Waste minimization, Sustainable development, Apparel manufacturing industry, Bayesian best-worst method

## Abstract

Industrial symbiosis, a promising approach for sustainable industrial practices, has garnered attention in recent days for its ability to enhance resource efficiency, minimize waste, and preserve the environment through collaborative exchanges among industries. In emerging economies like Bangladesh, integrating industrial symbiosis in the manufacturing industries offers the potential to balance economic growth with environmental sustainability. However, this integration encounters various barriers that complicate the implementation. Despite research on industrial symbiosis in robust economies, studies on emerging and developed economies are still scarce. To date, no research has yet investigated the barriers hindering the performance of industrial symbiosis in the Bangladeshi apparel manufacturing sector. To address this gap, this study integrates the Bayes theorem and the Best-Worst Method to identify and prioritize barriers to the Bangladeshi apparel manufacturing sector. From extensive literature reviews and expert validation, 17 barriers were identified. Findings reveal the “lack of technology and infrastructure readiness” as the most significant barrier, followed by “lack of inter-company cooperation” and “lack of management support”. Conquering these barriers empowers emerging economies to fortify the apparel manufacturing sector's resilience, resource efficiency, and environmental performance while fostering sustainable development via circular economy practices. This study is expected to guide policymakers and stakeholders in crafting targeted strategies for promoting steady growth and sustainable development in the apparel manufacturing sector of emerging economies like Bangladesh.

## 1 Introduction

Since the discovery of fire, human activity has progressively introduced pollutants into our environment, a trend that has dramatically intensified with the onset of the Industrial Revolution. As nations strive for economic growth, they inevitably rely on industrialization and urbanization, increasing environmental concerns [1]. For instance, a 1 % rise in industrial activity can result in a significant 11.8 % increase in CO_2_ emissions [2]. Balancing economic development without detrimentally impacting the environment is crucial. Industrial symbiosis (IS) offers a solution to this challenge. It involves the exchange of resources, energy, and by-products among industries, forming a network where waste from one industry becomes a resource for another. This concept has gained traction among policymakers and scholars over the past decade. The Intergovernmental Panel on Climate Change (IPCC) advocates IS as a strategy for sustainable growth and future industrial resilience [3]. Research indicates that 10.13039/100015147IS can support sustainable objectives while promoting economic prosperity [4,5]. Overcoming its challenges, IS can foster sustainable growth inspired by a circular economy [6], necessitating innovative collaboration and business models [7].

IS and circular economy are intertwined concepts that offer promising pathways toward sustainable resource management and economic development. Both concepts aim to decouple economic growth from resource consumption by promoting the reuse, recycling, and regeneration of materials [8]. IS aligns with the principles of circular economy, which have revitalized and shared knowledge in this domain with governmental and business leaders [9]. The approaches of industrial ecology, industrial symbiosis, and circular economy overlap considerably [10]. Moreover, initiatives such as the European Union's Circular Economy Action Plan emphasize IS's importance in achieving circularity and enhancing the sustainability of industrial systems [11].

Rapid industrial growth is essential for economic progress in developing nations but often leads to environmental degradation and resource depletion. These countries are at a critical point where they must reconcile industrial growth with sustainability. IS is especially relevant in these contexts, providing a viable pathway for sustainable industrial development. It encourages efficient resource use by enabling industries to share and recycle waste and by-products. This approach is especially pertinent in resource-constrained and environmentally-sensitive developing countries, aiding their transition to a sustainable and circular economy. It addresses ecological challenges, spurs innovation, opens new business avenues, and enhances global market competitiveness. Developed countries increasingly acknowledge IS's advantages in waste reduction, resource efficiency, and innovation promotion. For instance, the IS network in Sotenas, Sweden, has contributed to creating and maintaining jobs, launching new businesses, and significantly reducing CO_2_ emissions through resource sharing [5]. Similarly, Guiyang, China, has seen substantial CO_2_ emission reductions through urban IS implementation [12].

IS has become an essential strategy in the current industrial landscape, particularly for emerging economies facing the dual challenge of rapid industrialization and environmental sustainability. As a critical component of the circular economy, IS fosters collaborative waste-to-resource transformations between industries, reducing waste and environmental impact while enhancing resource efficiency and economic resilience. This is particularly crucial in emerging economies where industrial growth compromises ecological integrity. Various global implementations of IS have demonstrated its efficacy in lowering carbon footprints, conserving resources, and stimulating economic growth. Adopting IS can strategically benefit emerging economies like Bangladesh, where the apparel manufacturing sector (AMS) is a major economic driver. By integrating IS, these emerging economies can establish sustainable industrial models that balance economic growth with environmental conservation, setting an example for sustainable industrial development.

However, the potential of AMS for IS integration remains largely unexplored, especially in the context of emerging economies. Bangladesh is the second-largest ready-made apparel exporter after China [13]. The country generated approximately 42.61 billion US dollars from the AMS in FY 2021–22, accounting for 82 % of its total exports and employing around 4 million people [14]. In 2019, the AMS produced about 577,000 tonnes of apparel waste, potentially worth 100 million dollars if effectively utilized through IS networks [15]. Textile effluents, containing synthetic dyes and harmful chemicals, significantly impact Bangladesh's ecosystem, with many water quality parameters exceeding Environmental Quality Standards (EQS) [16]. Thus, IS could be a viable solution to these issues.

While there is substantial research on IS in developed countries, investigations tailored to emerging markets like Bangladesh remain scarce. Previous studies, such as Taqi et al. [17], have delved into the broader challenges Bangladesh's manufacturing sector faces without focusing on any specific industry. Similarly, Islam et al. [18] provided an overview of various approaches, opportunities, barriers, and policies impacting IS. Yet, their analysis lacked industry-specific insights and did not employ decision-making tools to dissect these complexities. Hence, there is a conspicuous gap in the literature concerning the barriers to implementing IS within Bangladesh's burgeoning industrial landscape. Driven by this observation, this research introduces a structured analytical framework to meticulously identify and analyze the barriers to IS adoption, particularly within the AMS of Bangladesh, thereby contributing novel insights to the field. This study, thereby, attempts to address the following research questions (**RQs**):

RQ1 What are the major barriers hindering the successful implementation of IS in the AMS of emerging economies?

RQ2 What is the hierarchical ranking among the identified barriers?

RQ3 How can the barriers be evaluated to achieve sustainable industrial practices?

To address the abovementioned RQs, this study aims to achieve the following research objectives (**ROs**):

RO1 To identify and comprehensively analyze the barriers hindering the successful implementation of IS in the AMS of emerging economies.

RO2 To effectively rank and prioritize the identified barriers to IS.

RO3 To provide evidence-based recommendations and insights to policymakers and practitioners to promote sustainable industrial practices through IS.

To meet the ROs of the study on IS practices in Bangladesh, a combined approach integrating the Bayes theorem with the Best-Worst Method (BWM) has been proposed. This methodology is critical in identifying, prioritizing, and assessing the barriers to adopting IS practices in Bangladesh. For the RO1, which concentrates on identifying and analyzing these barriers, the Bayesian BWM is particularly effective. It facilitates a detailed and nuanced examination of expert opinions and perceptions, going beyond mere identification to understand the complexities and variabilities of the barriers. Incorporating the Bayes theorem brings a probabilistic perspective, providing a more dynamic and accurate depiction of these barriers, which are essential in the context of Bangladesh, where various local economic, social, and environmental factors influence them. Moving to the RO2, the Bayesian BWM's strength in prioritizing barriers is evident. The method's capability to generate a context-relevant prioritization of barriers is crucial for formulating effective and targeted strategies to address them.

Overcoming barriers to IS in emerging economies holds transformative potential for economic growth and environmental sustainability. By effectively addressing these barriers, countries can harness IS to optimize resource use, turning waste from one production process into raw materials for another, thereby reducing costs and fostering innovation. This circular economy approach drives efficiency and competitiveness and opens up new markets and job opportunities, contributing significantly to economic development. Simultaneously, the environmental benefits of IS are profound. Reduced reliance on virgin materials diminishes environmental degradation and pollution. At the same time, the efficient use of resources cuts down on waste and greenhouse gas emissions, contributing to the fight against climate change. Moreover, IS promotes a more sustainable industrial model that can significantly reduce the ecological footprint of emerging economies, aligning their development trajectories with global sustainability goals. Thus, the dual focus on economic and environmental objectives creates a synergistic effect, where pursuing one reinforces the achievement of the other, offering a pathway to holistic and sustainable development in emerging economies.

The remainder of this study is organized as follows: Section 2 briefly reviews the literature. Section 3 discusses research design, applied techniques, and methodology. Section 4 presents the obtained results. Section 5 discusses the obtained results and the study's theoretical, managerial, and sustainability implications. Finally, section 6 concludes the study and discusses potential limitations and future research directions.

## 2 Literature review

This section reviews the present situation of IS in developed and emerging economies, related works, research gaps, and key barriers hindering the implementation of IS practices.

### 2.1 Industrial symbiosis in developed and emerging economies

IS has gained significant traction in developed nations as a pivotal approach towards achieving sustainable and resource-efficient economies. The present situation reflects a growing recognition of the interconnectedness between industries, where waste streams from one process serve as valuable inputs for another, fostering a closed-loop system. Collaborative networks and partnerships have flourished, bolstered by technological advancements, data analytics, and supply chain optimization. Governments and regulatory bodies in developed nations have increasingly embraced this concept, offering incentives and support to foster the implementation of 10.13039/100015147IS practices across sectors [19,20].

As these nations grapple with rapid industrialization and resource constraints, interconnecting industries to exchange waste and resources is gaining prominence. Despite challenges like limited technological infrastructure and regulatory frameworks, collaborative networks are emerging to facilitate resource sharing and waste reduction. In Bangladesh, the current landscape of IS is undergoing gradual transformation, reflecting an increasing recognition of its potential to address pressing environmental and economic challenges. As a developing nation with a rapidly growing industrial sector, the concept of connecting industries to optimize resource utilization and reduce waste is gaining attention. However, the journey faces several barriers that need to be addressed.

### 2.2 Related works, research gap and contributions

Over the decades, IS has gained much attention from researchers and academicians, and many studies have been conducted on it. In developed economies, the implementation of IS has shown significant improvement. In the Västra Götaland Region of Sweden, the symbiotic network between the mushroom and beer companies has gained significant economic and environmental benefits [21]. In Europe, three critical elements, such as the type of waste stream or by-product, transportation costs, and the market value of secondary products, affect whether IS is implemented successfully [20]. Yu et al. [22] suggested many frameworks for implementing 10.13039/100015147IS in the Dutch construction sector, including the implementation of stringent waste classifications on-site, the creation of an information-sharing platform to enhance business communication, financial support for up-cycling technology innovation, and the incorporation of circular business models to increase the space for collaboration.

The emerging nations are also marching forward to utilize the auspicious impact of IS. The symbiosis network of Yongcheng, China, has reduced 0.43 Metric tons (Mt) of municipal solid waste and 4.88 Mt of CO_2_ emission while saving 1.07 Mt of coal, 17 % of energy [23]. Bacudio et al. [24] identified and analyzed the barriers to 10.13039/100015147IS in the Philippines, where the dearth of top management support, absence of policy to incentivize the initiative of 10.13039/100015147IS, and lack of funding to promote 10.13039/100015147IS were found to be the most pressing barriers. The adoption of IS in Liuzhou City, China, resulted in annual reductions of 2.3 million tons of CO_2_ emissions, 6.9 million tons of solid waste, and 204.7 million tons of ore mining [25].

Henriques et al. [26] identified the enablers and barriers of IS implementation using Sectoral Analysis, where the factors are categorized into several sectors, such as social, economic, environmental, geographical, and policy. Golev et al. [27] assessed the IS barriers using the Maturity Grid, which included five steps, where Stage 1 implies that IS is not recognized. Stage 5 indicates that all the stakeholders collaborate and trust to form a desirable future. To transform a symbiotic network from Stage 1 to Stage 5, environmental regulation, lack of cooperation and trust among industries, and lack of information sharing were identified as major barriers. Neves et al. [28] and Zhang et al. [29] identified the barriers through a literature review. However, qualitative assessment has accomplished these studies without incorporating Multi-Criteria decision-making (MCDM) tools.

Yang et al. [30] utilized the Analytical Hierarchy Process (AHP) and Technique for Order Preference by Similarity to Ideal Solution (TOPSIS) to evaluate the IS barriers, which found that technological, economic, and safety barriers are the most significant barriers. However, their study didn't show any interrelationship among the barriers. Bacudio et al. [24] applied the Decision-Making Trial and Evaluation Laboratory (DEMATEL) approach to identifying cause-effect relationships among the barriers to IS implementation. This study reveals that *lack of top management* support is the most significant cause barrier*,* and *lack of information sharing* is the most significant effect barrier. However, the main limitation of DEMATEL is that it ranks alternatives based on their interdependencies, but other factors are not considered throughout the decision-making process [31]. Table 1 presents a summary of the recent studies on IS implementation.

**Table 1:** Recent studies on IS implementation.

Source	Focused sector	Objectives	Applied tools
Yang et al. [30]	Manufacturing industries	To evaluate the barriers to IS implementation of an industrial park.	AHP-TOPSIS
Taqi et al. [17]	Manufacturing industries	To investigate the barriers to IS implementation.	DEMATEL
Cárcamo & Peñabaena-Niebles [19]	Waste management sector	To identify the opportunities and challenges of IS implementation.	Review paper
Sellitto et al. [32]	Brazilian manufacturing industries	To identify the barriers, drivers, and relationships in IS network.	Descriptive statistics
Henriques et al. [26]	Manufacturing and production industries	To analyze the enablers and barriers to IS implementation.	Comprehensive assessment with sectoral analysis
Kosmol & Otto [33]	Manufacturing industries	To perform a qualitative content analysis of the barriers to IS implementation.	PRISMA
Neves et al. [34]	Business organizations	To analyze the present condition, upcoming challenges, and prospects of IS in Portugal.	Review paper
Ferreira et al. [35]	Agriculture industry	To map the IS network of the biomass fluidized bed boiler.	Value stream mapping (VSM)
Baldassarre et al. [7]	Manufacturing industries	To design the process for eco-industrial parks.	Descriptive statistics
Martin & Harris [5]	Manufacturing and business organizations	To reveal the environmental and socioeconomic implications of the IS network.	Life cycle assessment
Mantese & Amaral [36]	Manufacturing and business organizations	To comparatively evaluate the IS indicators.	Simulation through an agent-based model
de Abreu & Ceglia [6]	Manufacturing industries	To investigate how developing institutional capacity through IS can contribute to the creation of a circular economy.	Content analysis to make inferences from the interviews
Daddi et al. [4]	Manufacturing industries	To measure the environmental benefits of IS implementation.	Life cycle assessment
Boons et al. [37]	Manufacturing industries	To propose a conceptual and theoretical framework for analyzing the barriers to IS implementation.	Comparative analysis through literature review.
Bacudio et al. [24]	Manufacturing and business organizations	To identify the barriers to utilizing the synergy of IS implementation.	DEMATEL

Despite the advancements made, there remain several gaps within the realm of research that demand attention. A noteworthy aspect lacking significant exploration pertains to the recognition and comprehensive evaluation of barriers hindering the adoption of IS within the apparel manufacturing industry. This aspect has yet to receive substantial investigation, especially from the context of emerging economies like Bangladesh. The proposed study addresses this gap by incorporating the Bayes theorem and the Best-Worst Method. The comprehension of barriers tied to the implementation of IS holds pivotal importance for many stakeholders, including policymakers, investors, and industry practitioners. This knowledge is crucial for fostering sustainable practices and effectively advancing towards attaining the Sustainable Development Goals (SDGs) within emerging economies.

This study endeavor introduces several significant contributions to the existing body of knowledge. Firstly, it adds value to the current scholarly discourse by meticulously pinpointing and undertaking a comprehensive analysis of the barriers that impede the effective integration of IS within the AMS of an emerging economy. This endeavor holds the potential to provide emerging economies with insights that are instrumental in successfully embracing IS practices. Secondly, a notable advancement lies in the prioritization of these barriers. This study takes a stride ahead by incorporating both the Bayes theorem and the sophisticated BWM, thus presenting a more contemporary approach. This represents the pioneering instance wherein Bayesian BWM, an emerging economy context, and barriers connected to IS adoption are amalgamated within a singular framework.

Moreover, the study extends its impact by providing recommendations and insights grounded in empirical evidence. These recommendations serve as a practical guide for policymakers and practitioners in effectively devising strategies to address the identified prioritized barriers. Furthermore, these strategies are envisioned to facilitate the promotion of sustainable industrial practices, including the adoption of circular economy principles through IS integration. Lastly, the anticipated outcomes of this study carry substantial weight in contributing to the achievement of various SDGs, most notably Goal 12 (Responsible Consumption and Production), Goal 13 (Climate Action), and Goal 15 (Life on Land), among others.

### 2.3 Key barriers to successful implementation of IS

An extensive research endeavor was undertaken to identify the primary barriers affecting the implementation of IS within the specific context of Bangladesh. This effort encompassed a comprehensive search across well-established databases such as Scopus, ScienceDirect, and Google Scholar. The research was specifically geared towards exploring a range of pivotal terms, including “Industrial Symbiosis,” “Emerging Economy,” “Barriers/Impediments/Challenges,” and “Sustainable Practices,” among others. This meticulous examination yielded 18 distinct barriers to the successful implementation of IS. Subsequently, to ensure their applicability within the landscape of the Bangladeshi AMS, these identified barriers were subjected to validation through consultations with pertinent subject matter experts. This collaborative process established a refined selection of 17 pertinent barriers. These chosen barriers were further categorized into four distinct groups. Details of the finalized 17 barriers are listed in Table 2.

**Table 2:** Details of the finalized barriers based on literature review and expert feedback.

Barriers to IS with the code	Description	Sources
**C1. Economic barriers**		
EB1. High processing cost	The cost associated with fully processing a by-product unit.	Sellitto et al. [32]; Shen et al. [38]
EB2. High logistics cost	Cost of storage and transportation of an IS unit.	Ferreira et al. [35]
EB3. Disruption of availability	Excessive or lack of availability creates an imbalance between the generation and consumption of by-products.	Expert opinion
EB4. Lack of funding to promote IS	Insufficient funding has been allocated to advance IS and disseminate knowledge.	Henriques et al. [26]
EB5. Market immaturity	The market is not adequately prepared for the incorporation of IS.	Henriques et al. [26]
**C2. Management related barriers**		
MB1. Lack of management support	Industrial facilities' current management practices do not yet incorporate the IS approach into their policies.	Kosmol & Otto [33]; Bacudio et al. [24]
MB2. Lack of inter-company cooperation	Companies play an insufficient role in promoting IS in terms of integration and information sharing.	Marra et al. [39]
MB3. Lack of trust among the locators	At the initiation phase, organizations must be compliant in order to manage the cooperative mechanism.	Neves et al. [34]; Walls & Paquin [40]
MB4. Personal barriers	Managers are not aware of the opportunities to reuse by-products and waste.	Sellitto et al. [32]
**C3. Cognitive and Technological barriers**		
CT1. Lack of research and groundwork	Current management practices in industrial plants have yet to include the IS approach as part of their policy.	Yang et al. [30]; Boons et al. [37]
CT2. Lack of awareness of the IS concept	Companies are not informed of existing opportunities for reusing by-products.	Bacudio et al. [24]
CT3. Lack of technology and infrastructure readiness	Necessary technology and infrastructure are not available for sustainable by-product exchange.	Bacudio et al. [24]
CT4. Economic and technological infeasibility	The adoption and management of technology is not economically viable.	Kosmol & Otto [33]; Debnath et al. [41]
**C4. Environmental and Policy Barrier**		
EP1. Lack of legal requirements	Companies are not capable of complying with the local rules and legislation.	Henriques et al. [26]
EP2. Lack of policy to incentivize IS	Government policies are not put in place to stimulate and regulate IS.	Yang et al. [30]
EP3. Ecological Safety barrier	Potential safety for ecology impedes policymakers, adoption of IS.	Yang et al. [30]
EP4. Low waste disposal cost	Low waste disposal costs encourage companies to dump or landfill instead of reusing.	Henriques et al. [26]; Kosmol & Otto [33]

## 3 Methods

This study investigates the prominent barriers that might impede the adoption of IS and sustainability within Bangladesh's AMS. Integrating IS can enhance resource efficiency, curtail waste generation, and elevate environmental performance. While certain individual companies may have initiated efforts to enhance resource efficiency and sustainability, widespread implementation of formal IS practices across the entire AMS has not been extensively documented. Hence, this sector has been selected as a pertinent case study to delve into the primary barriers that hinder the effective implementation of IS practices.

### 3.1 Survey design

This study uses a two-stage approach to gather data on IS practices in green apparel manufacturing. The focus is on companies in Bangladesh that have adopted green manufacturing principles and hold Leadership in Energy and Environmental Design (LEED) certification from the US Green Building Council. These companies are more enthusiastic about effective waste and by-product management [42]. As the Bangladesh Garment Manufacturers and Exporters Association (BGMEA) reported, 135 green apparel manufacturing factories nationwide are certified by LEED and categorized into Platinum, Gold, and Silver classes [43].

In the first stage, we identified a list of barriers to IS from the literature, which we then validated with experts from LEED-certified factories. These barriers were evaluated based on their relevance to IS practices in the local context. We sought feedback using a simple 'yes' or ‘no’ questionnaire (see Appendix A) to determine which barriers were most pertinent, eliminating any that lacked consensus or were redundant. Barriers that garnered most of the 'yes' votes from the experts were selected for further analysis.

**Fig. 1:**
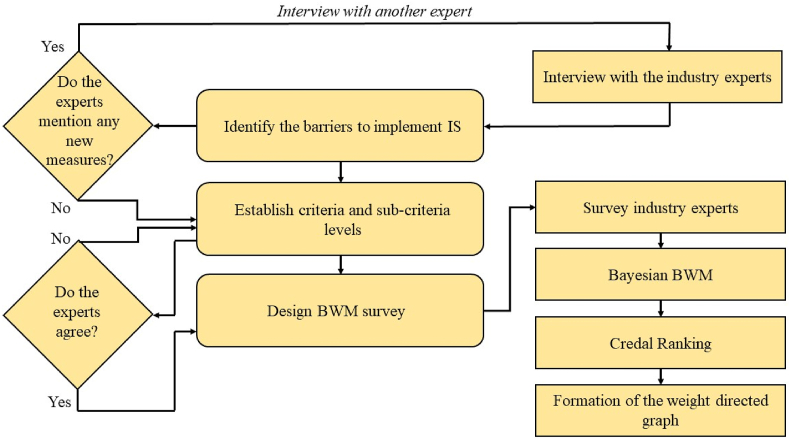
Workflow of the study.

In the validation phase, the experts eliminated two barriers (“Excessive Supply” and “Shortage of Supply”) and added one additional barrier (“Disruption of Availability”). The final selected list of seventeen barriers is provided in Table 2. These experts verified and finalized the list, organizing the barriers into four groups. We developed a BWM survey for the second stage to deepen our understanding of these barriers [44,45]. Industry experts ranked the most to least significant barriers and provided detailed comparisons between them. The responses were analyzed using the Bayesian BWM method to ensure precise and reliable results. The overall workflow of this study is depicted in Fig. 1.

Compared to conventional Likert scale surveys, surveys utilizing the BWM and MCDM techniques exhibit higher complexity [15,46]. Unlike conventional Likert scales, this study employs an advanced survey technique to explore barriers to IS practices in Bangladesh's apparel sector. From February 10, 2023, to June 10, 2023, digital surveys were sent to professionals at 135 LEED-certified factories. Over 50 companies were specifically targeted to ensure a broad industry representation, including 35 silver, 7 gold, and 8 platinum-class LEED-certified factories.

Using purposive sampling, we targeted individuals with at least five years of relevant experience and significant roles within the apparel manufacturing sector. This approach ensures the collection of precise and relevant data from experts who are well-informed about the subject matter [47,48]. This method entails carefully choosing individuals or a group of individuals from a larger population based on their unique attributes, expertise, and relevance to the research topic [49,50]. Of the 50 companies approached, 19 initially responded: nine from the 35 silver-certified companies, six from the seven gold-certified companies, and four from the eight platinum-class LEED-certified companies. After a careful screening process to verify the data's validity and relevance, 12 responses were deemed suitable for further analysis. Exclusions were made for responses that showed duplication, lack of logical consistency, or insufficient relevance to the sector's specific needs. This stringent selection process ensured that the data used in the study came from respondents who were adequately knowledgeable and directly engaged in pertinent roles within the green apparel manufacturing context.

Although the sample size of 12 decision-makers (DMs) in our study may seem small, it was carefully chosen to align with the qualitative research approach, focusing on depth and expertise rather than quantity. Each DM was selected for their extensive experience in the AMS, ensuring they could provide informed insights into the barriers to implementing IS practices. The Bayesian BWM method, known for its probabilistic modeling, adds to the reliability of our findings by effectively managing uncertainties and expert judgment variabilities. This approach has been validated by prior studies [[51], [52], [53]]. Therefore, the study's valid and reliable results offer meaningful insights into IS practices within a large and diverse industry [54]. A summary of the participating respondents has been presented in Table 3. Eleven out of the twelve respondents' companies hold ISO 9000 certification. This pattern indicates that the sampled businesses have already implemented quality management systems and are actively pursuing the adoption of a green manufacturing strategy.

**Table 3:** A summary of the survey respondents.

SN.	Company Age (Years)	Company Size	ISO 9000 Certification	Exp.	Expertise	Education
1	25	Large	Yes	6	Production and Operations	Bachelor Degree
2	12	Large	Yes	6	Industrial Engineering	Bachelor Degree
3	18	Large	Yes	6	Supply Chain Management	Master's Degree
4	15	Large	Yes	5	Manager Quality Control	Bachelor Degree
5	15	Medium	Yes	5	Planning and Coordination	Bachelor Degree
6	20	Large	No	5	Quality Control and Management	Bachelor Degree
7	18	Large	Yes	10	Industrial Engineering	Master's Degree
8	20	Large	Yes	6	Supply Chain Management	Bachelor Degree
9	23	Large	Yes	12	Planning and Coordination	Master's Degree
10	12	Large	Yes	6	Supply Chain Management	Bachelor Degree
11	15	Large	Yes	7	Industrial Engineering	Master's Degree
12	20	Large	Yes	6	Supply Chain Management	Bachelor Degree

Small: 1 to 100; Medium: 101 to 1000; Large: More than 1000 employees and workers.

### 3.2 Bayesian Best-Worst Method

The Bayesian BWM is an advanced MCDM technique that builds on the linear BWM framework [46]. BWM, as established by Rezaei [54], is recognized for its efficiency in handling decision-making processes by requiring decision-makers to identify the most and least favorable factors from a set and to conduct pairwise comparisons. This method stands out in the MCDM landscape for its streamlined approach, which significantly reduces the number of necessary comparisons. Traditional BWM facilitates decision-making with a more focused set of comparisons, requiring only 2n-3 evaluations [55] as opposed to the n(n-1)/2 comparisons demanded by methods like AHP [56,57]. However, traditional BWM encounters limitations when aggregating insights from multiple experts [15] since linear BWM processes individual expert weights through averaging, a step that may dilute the specificity of expert insights and introduce the risk of information loss.

To address these challenges, in our study, we integrated the Bayes theorem with traditional BWM, as introduced by Mohammadi & Rezaei [58]. This integration allows for a probabilistic interpretation of decision-making factors, treating each as a random event and assigning weights according to their corresponding probabilities. This probabilistic approach enables a more nuanced and accurate analysis of uncertainties inherent in decision-making processes. This study uses the following steps to apply the Bayesian BWM approach [51,58].

Step 1 Criteria Selection

Each expert selects the ‘Best’ (B) and ‘Worst’ (W) criteria from a set $C=\left\{c_{1},c_{2},c_{3},c_{4},\ldots \ldots \ldots .\;c_{m}\right\}$, where ($C_{B}^{n})$ and ($C_{W}^{n})$ represent the best and worst criteria, respectively, as identified by expert *n*. This step is crucial for establishing the framework within which pairwise comparisons will be conducted.

Step 2 Pairwise Comparison of ‘Best’ to Others

Experts assign a value from 1 to 9 to express their preference for the ‘Best’ criterion over each of the others in set *C*, forming the Best-to-Others vector, $V_{B}^{n}$. This vector facilitates a quantifiable comparison of the selected ‘Best’ criterion against all others, based on expert evaluations. The vector Best-to-Others ($V_{B}^{n}$) is calculated by comparing each feature of expert n to the features of all other experts. The results of these comparisons are then used to create a vector that indicates which features experts believe are most important.

(1) $$V_{B}^{n}=\left(v_{B1}^{n}{,v}_{B2}^{n},v_{B3}^{n}{,\ldots \;\ldots \;\ldots ,v}_{Bm}^{n}\right);\;n=1,2,3,\ldots \;\ldots \;\ldots ,L$$

Where $v_{Bj}^{n}$ represents the preference of the “best” criterion ($C_{B}^{n})$ over each criterion $c_{j}\in C$ for expert *n*. The comprehensive lists of all formed Best-to-Others vectors for the main clusters and sub-clusters are provided in Table B1 to Table B5 of Appendix B in the supplementary materials.

Step 3 Pairwise Comparison of Others to ‘Worst'

Similarly, experts compare each criterion to the ‘Worst’ using a 1 to 9 scale, resulting in the Others-to-Worst vector, $V_{W}^{n}$. This comparison inversely evaluates the relative importance of each criterion against the ‘Worst’. The Others-to-Worst vector $\left(V_{W}^{n}\right)$ results from the set of comparisons for expert n as follows:

(2) $${\left(V_{B}^{n}=\left(v_{1W}^{n}{,v}_{2W}^{n},v_{3W}^{n}{,\ldots \;\ldots \;\ldots ,v}_{mW}^{n}\right)\right)}^{T}$$

Where, $v_{jW}^{n}$ represents the preference $c_{j}\in C$ over the worst criterion ($C_{B}^{n})$ for expert *n*. The comprehensive lists of all formed Others-to-Worst vectors for the main clusters and sub-clusters are provided in Table C1 to Table C5 in Appendix C in the supplementary materials.

Step 4 Computing aggregated weights

In this phase, the Bayesian BWM utilizes a probabilistic model to aggregate the weights assigned by all experts, effectively integrating individual assessments into a coherent decision-making framework. This process involves the calculation of both the collective weights (denoted as $\omega^{*})$ for all criteria and the individual weights $\left(\omega^{n}\right)$ for each expert. The methodology unfolds as follows:

(3) $$V_{B}^{n}|\omega^{n}∼multinomial\;\left(\frac{1}{\omega^{n}}\right),\forall_{n}=1,2,3,\ldots \;\ldots \;\ldots ,L$$

(4) $$V_{W}^{n}|\omega^{n}∼multinomial\;\left(\omega^{n}\right),\forall_{n}=1,2,3,\ldots \;\ldots \;\ldots ,L$$

(5) $$\omega^{n}|\omega^{*}∼Dir\;\left(\gamma \times \omega^{*}\right),\forall_{n}=1,2,3,\ldots \;\ldots \;\ldots ,L$$

(6) γ∼gamma(0.1,0.1)

(7) $$\omega^{*}∼Dir$$

The multinomial distribution is represented by the term “multinomial,” while the gamma distribution with parameters set at 0.1 is denoted by “*Gamma* (0.1, 0.1)," and " *Dir* " signifies the Dirichlet distribution. We can navigate through the complexities of probabilistic models by utilizing Markov-chain Monte Carlo (MCMC) sampling, a method essential for solving problems lacking straightforward analytical solutions [51]. The implementation of Bayesian BWM is carried out in JAGS (Just Another Gibbs Sampler), a tool that is both freely available and open-source [59]. Through this approach, we derive a series of samples, S, from the posterior distributions of the collective weights, $\omega^{*}$, as per the Bayesian BWM methodology, this process of weight aggregation allows for a deeper analysis of the relative importance of each criterion, facilitating a nuanced comparison based on Bayesian inference. The subsequent definitions and calculations provide a framework for understanding these aggregated priorities and their implications in a probabilistic context based on $\omega^{*}$.

Definition 1 In the context of comparing two criteria, $c_{i}$ and $c_{j}$, the concept of credal ordering, as introduced by Mohammadi & Rezaei [58], is denoted by *O*, and defined as $O=(c_{i}$ , $c_{j}$ , R, d*).* Here, R specifies the relationship in terms of performance between $c_{i}$ and $c_{j}$, (such as <, >, or =), and *d*, which ranges from 0 to 1, represents the confidence level in this relational assessment.

Definition 2 Credal ranking encompasses a collection of credal orderings for every possible pair of criteria within the set $C=c_{1},c_{2},c_{3}\;\ldots \;\ldots \;\ldots ,c_{n}\;)$, as outlined by Mohammadi & Rezaei [58]. This set forms a comprehensive framework for evaluating and ranking all criteria based on their relative importance and performance.

To quantify the degree of preference between any two criteria $c_{i}$ and $c_{j}$, we employ a series of samples, *S*, derived from JAGS. The calculation of preference is represented as $P\;\left(c_{i}>c_{j}\right)\text{,}$ is determined by the average number of instances where the weight of $c_{i}$ exceeds that of $c_{j}$ across all samples, *S*, which can be mathematically expressed as:

(8) $$P\;\left(c_{i}>c_{j}\right)=\frac{1}{S}\sum_{s=1}^{S}{(\omega }_{i}^{*S}>\omega_{j}^{*S})$$

This approach provides a probabilistic basis for ranking criteria, leveraging Bayesian inference to discern the relative importance of each criterion within the decision-making framework.

## 4 Results

This section presents the findings of the Bayesian BWM applied to assess the hierarchical ranking of the barriers to IS implementation in Bangladeshi AMS. The outcomes are derived following the detailed step-by-step process of the Bayesian BWM, as outlined in the preceding section. For each cluster and sub-cluster, a weighted directed graph is constructed, and the nodes of the graph represent the factors with average weights computed from the mean of $\omega^{*}$ distribution. $A\overset{d}{\to }B$ means that A is preferable to B with a confidence degree of *d*. In other words, the entire graph depicts the credal ranking of a set of criteria, and each edge indicates the credal ordering.

**Fig. 2:**
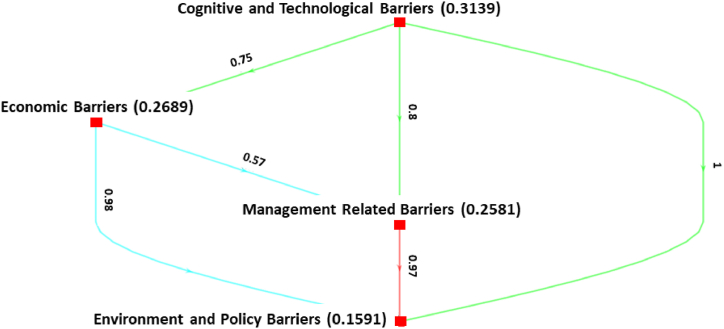
Ranking of the major clusters.

Fig. 2 presents the weights of the main clusters. Among the four clusters, *cognitive and technological barriers* (0.3139) are identified as the most important barriers, followed by *economic barriers* (0.2689), *management-related barriers* (0.2581), and *environmental and policy barriers* (0.1591). *Cognitive and technological barriers* are more important, with a 0.75 confidence level than *economic barriers*, with a 0.80 confidence score than *management-related barriers*, with a confidence level of 1 than *environmental and policy barriers*. *Economic barriers* are more important, with 0.57 confidence than *management-related barriers*, with 0.97 confidence than *environmental and policy barriers*. *Management-related barriers* are more important, with 0.97 confidence, than *environmental and policy barriers*. Although the confidence levels are calculated based on experts' preferences, we consider a 0.60 threshold to indicate strong confidence [59].

**Fig. 3:**
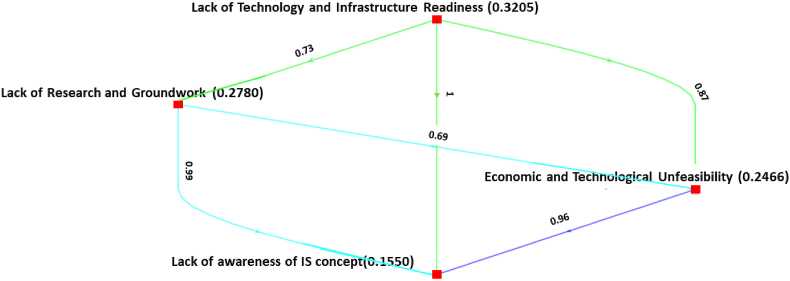
Ranking of cognitive and technological barriers.

Moreover, the local ranking of *cognitive and technological barriers* is presented in Fig. 3. It reveals that *lack of technology and infrastructure readiness* (0.3205) is considered the most important barrier, with a confidence level of 0.73, then *lack of research and groundwork* (0.2780), with a confidence level of 0.87 compared to *economic and technological infeasibility* (0.2466), and a confidence level of 0.69 compared to *lack of awareness of IS concept* (0.1550). Here, the confidence degree of 0.69 indicates most experts consider *a lack of technology and infrastructure readiness as more important; however, a significant number of experts consider a lack of awareness of the IS concept* to be more important.

**Fig. 4:**
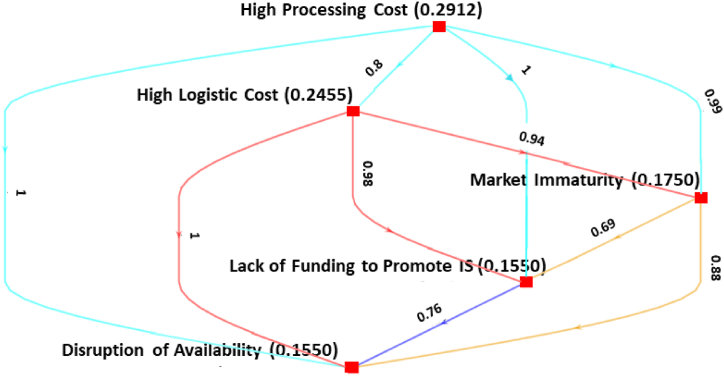
Ranking of economic barriers.

The credal ranking for *economic barriers* in Fig. 4 shows that the sub-criterion of *high processing cost* (0.2912) is considered the most important, with a confidence score of 0.80 or more than the other sub-criteria. The next three most prioritized measures are *High logistics cost* (0.2455), *market immaturity* (0.1750), and *lack of* funding *to promote IS* (0.1563). *Disruption of availability* (0.1320) is considered the least important measure. It shows that *high processing cost* is more important than *disruption of availability* and *market immaturity*.

**Fig. 5:**
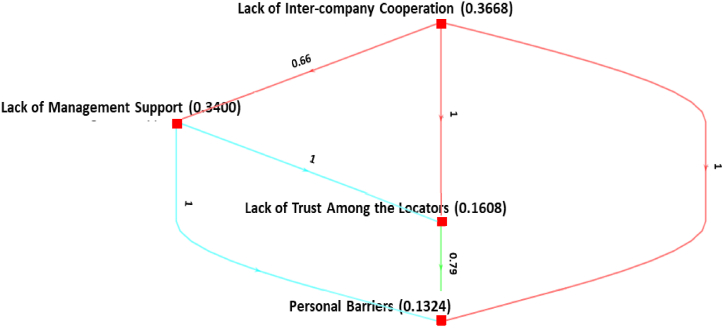
Ranking of management-related barriers.

Concerning the *Management-related barriers*, the barrier *lack of inter-company cooperation* (0.3668) appears to be the most important, followed by *lack of management* support (0.3400), *lack of trust among the locators* (0.1608), and *Personal barriers to initiate IS* (0.1324) as shown in Fig. 5. *Lack of inter-company cooperation* is more important with an absolute degree of confidence than lack of trust among the locators and personal barriers to initiating *IS*.

**Fig. 6:**
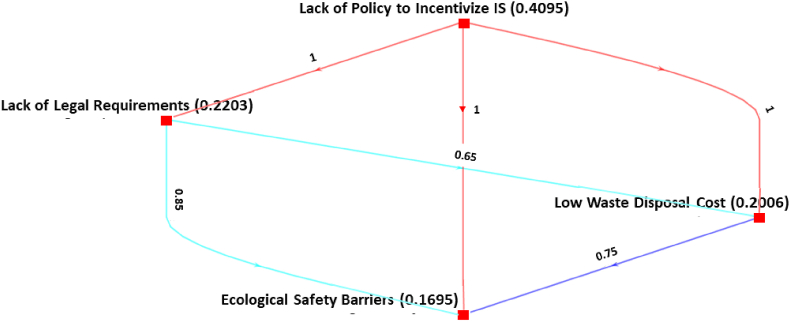
Ranking of environmental and policy barriers.

Concerning the *environmental and policy barriers*, Fig. 6 explicitly shows that the barrier *lack of policy to incentivize IS* (0.4095) is superior to the other two barriers named *lack of legal requirements* (0.2203) and *low waste disposal cost* (0.2006) with a confidence score of 1. Although *environmental and policy barriers* are important to preserve our ecology, technological, economic, and management-related barriers are more influential in the present situation of the AMS in emerging economies.

The global rankings of 17 barriers are calculated by multiplying the cluster-level weights by the corresponding local weights for each barrier.

**Fig. 7:**
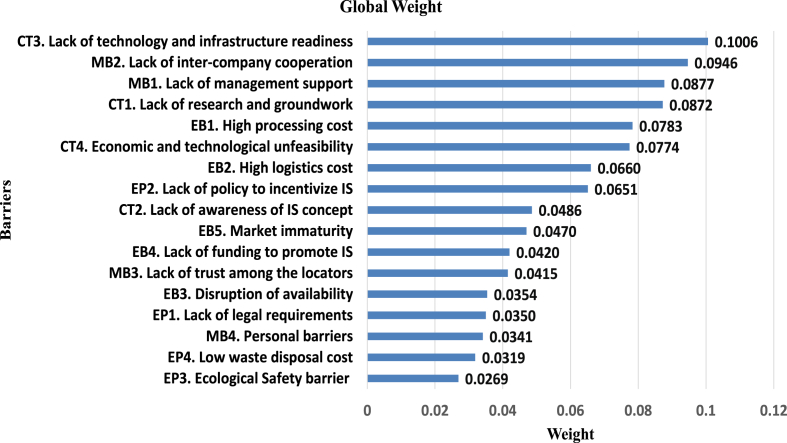
Global ranking of the barriers.

Fig. 7 showcases a graphical depiction of the worldwide ranking of barriers to adopting IS in the AMS in Bangladesh, which has been obtained from Table 4.

**Table 4:** The local and global weights of criteria and sub-criteria.

Barriers	Local Weight	Global Weight
***C1. Economic barriers***	0.2689	–
EB1. High processing cost	0.2912	0.0783
EB2. High logistics cost	0.2455	0.0660
EB3. Disruption of availability	0.1320	0.0354
EB4. Lack of funding to promote IS	0.1563	0.0420
EB5. Market immaturity	0.1750	0.0470
***C2. Management related barriers***	0.2581	–
MB1. Lack of management support	0.3400	0.0877
MB2. Lack of inter-company cooperation	0.3668	0.0946
MB3. Lack of trust among the locators	0.1608	0.0415
MB4. Personal barriers	0.1324	0.0341
***C3. Cognitive and Technological barriers***	0.3139	–
CT1. Lack of research and groundwork	0.2780	0.0872
CT2. Lack of awareness of IS concept	0.1550	0.0486
CT3. Lack of technology and infrastructure readiness	0.3205	0.1006
CT4. Economic and technological unfeasibility	0.2466	0.0774
***C4. Environmental and Policy barriers***	0.1591	–
EP1. Lack of legal requirements	0.2203	0.0350
EP2. Lack of policy to incentivize IS	0.4095	0.0651
EP3. Ecological Safety barrier	0.1695	0.0269
EP4. Low waste disposal cost	0.2006	0.0319

Table 4 displays the local and global weights for all barriers, indicating that the most substantial barrier is the *lack of technology and infrastructure readiness* (0.1006). Conversely, the least significant barrier identified is the *ecological safety barrier* (0.0269). Table 5 provides the confidence scores for the barriers within all clusters [60,61].

**Table 5:** Cluster level confidence score.

Cluster	C1	C2	C3	C4
C1. Economic barriers	0	0.5691	0.2522	0.9774
C2. Management related barriers	0.4309	0	0.202	0.9674
C3. Cognitive and Technological barriers	0.7478	0.798	0	0.9954
C4. Environmental and Policy barriers	0.0226	0.0326	0.0046	0

Again, Table 6 provides the confidence scores for the barriers within all sub-clusters, offering detailed insights into their relative significance.

**Table 6:** Confidence scores of all sub-clusters barriers.

Economic barriers	EB1	EB2	EB3	EB4	EB5
EB1. High processing cost	0	0.8033	0.9996	0.9971	0.9906
EB2. High logistics cost	0.1966	0	0.9958	0.978	0.9404
EB3. Disruption of availability	0.0004	0.0042	0	0.2388	0.1155
EB4. Lack of funding to promote IS	0.0029	0.022	0.7612	0	0.3119
EB5. Market immaturity	0.0094	0.0596	0.8845	0.6881	0

***Cognitive and Technological barriers***	***CT1***	***CT2***	***CT3***	***CT4***
CT1. Lack of research and groundwork	0	0.9852	0.2716	0.6874
CT2. Lack of awareness of IS concept	0.0148	0	0.0031	0.0418
CT3. Lack of technology and infrastructure readiness	0.7284	0.9969	0	0.8654
CT4. Economic and technological unfeasibility	0.3126	0.9582	0.1346	0

***Management related barriers***	***MB1***	***MB2***	***MB3***	***MB4***
MB1. Lack of management support	0	0.344	0.9995	1
MB2. Lack of inter-company cooperation	0.656	0	0.9998	1
MB3. Lack of trust among the locators	0.0005	0.0002	0	0.7903
MB4. Personal barriers	0	0	0.2097	0

***Environmental and Policy Barrier***	***EP1***	***EP2***	***EP3***	***EP4***
EP1. Lack of legal requirements	0	0.004	0.8517	0.6485
EP2. Lack of policy to incentivize IS	0.996	0	0.9998	0.9989
EP3. Ecological Safety barrier	0.1483	0.0002	0	0.2544
EP4. Low waste disposal cost	0.3515	0.0011	0.7456	0

## 5 Discussion

This study aims to investigate and rank the barriers to implementing IS in the context of AMS in Bangladesh. The barriers are clustered into four groups and assessed using the Bayesian BWM to carry out the result. The global ranking of all sub-criteria is exhibited in Fig. 7. The global ranking of sub-criteria indicates that *lack of technology and infrastructure readiness* (0.1006) is the most pressing barrier, which is in line with the findings of Yang et al. [30]. Recycle, recovery, and evaluation technology are crucial in establishing IS relationships [30]. However, the result differs from the findings of Bacudio et al. [24] because the study was done in small and medium-sized enterprises (SMEs) where sophisticated technology is less needed. According to industry experts, having the latest technologies to recycle waste and by-products is crucial. However, emerging countries are yet to develop the necessary technology.

Moreover, the second and third most influential barriers are *Lack of inter-company cooperation* (0.0947) and *Lack of management* support (0.0878) from the cluster of *Management-related* barriers, which indicates that after acquiring the necessary technology, it is important to establish a robust network to utilize the synergy among industries. Taqi et al. [17] asserted that the *lack of inter-company cooperation* complicates the journey of IS [17]. To foster a strong network of IS, the government, industry, and stakeholders have to act in a positive feedback loop [40]. However, *lack of management* support significantly impedes the other factors as top management motivates employees, develops new ideas, and allocates resources [32].

Three economic barriers, named *high processing cost* (0.0783), *economic and technological infeasibility* (0.0774), and *high logistic cost* (0.066), have ranked fifth, sixth, and seventh, respectively, among the sub-criteria. Sellitto et al. [32] also identified high processing and logistics costs as a prime barrier to the Brazilian manufacturing sector. Excessive logistics costs hampers the transfer of waste and by-products, whereas high processing cost makes the transformation operations infeasible [32].

The weighted value of the *lack of policy to incentivize IS* found to be 0.0487, which ranked eighth in the global ranking list. The role of policy in supporting 10.13039/100015147IS has been emphasized by some researchers like Boons et al. [37] and Walls & Paquin [40]. Fostering the development of any environment-supportive measure often requires implementing conducive policies, including subsidies and low-interest loans [62]. The primary factors that policy barriers are important are that some initiatives like tax cuts and refund policies taken by the government always work as agents to harness the synergy among the industries [63]. Another economic barrier, disruption of availability, exhibits excessive or shortage of supply, creating an imbalance between availability and demand, which is in line with the findings of Madsen et al. [62].

*Low waste disposal cost* (0.0319) and *lack of legal requirements* (0.0350) are interrelated barriers. The concept of *low waste disposal cost* suggests that companies or individuals may not face significant financial burdens when disposing of waste, which could lead to less incentive to invest in sustainable waste management practices [22]. In this context, it is necessary to establish the legal requirements from the state where the industry stakeholders are encouraged to recycle the waste to gain a competitive advantage [64]. According to the apparel industry professionals, since emerging countries' legal requirements and state laws are not so hard and fast, Low waste disposal costs and Ecological safety barriers have stood at the bottom of the global ranking list. Thus, *environment and policy-related barriers* gained less priority than the other criteria.

Overcoming the significant barriers to IS has significantly benefited various developing nations. For instance, according to Lu et al. [23], after the successful implementation of IS, the symbiotic network of Yongcheng, China, saved 2.37 metric tons (Mt) of slag substitution, 0.43 Mt municipal solid waste, and 4.88 Mt CO_2_ emission. Through the application of IS and circular economy (CE) principles, the Italian paper manufacturing company ‘Favini’ has significantly reduced the use of virgin material, reducing its raw material costs [65]. Implementing CE and IS programs allowed Aquafil and Itelyum to obtain EU financing, which allowed them to start new eco-innovation projects [66].

### 5.1 Theoretical implications

The study contributes by enhancing the academic and practical understanding of the integration process of IS in the apparel manufacturing industry, which has not been explored yet, particularly for an emerging economy like Bangladesh. By systematically identifying and ranking the barriers to integration, the study holds the potential to provide emerging economies with insights that are instrumental in successfully embracing IS practices.

This study takes a stride ahead by incorporating both the Bayes theorem and the sophisticated BWM, thus presenting a more contemporary approach. As far as we know, this represents a pioneering instance where Bayesian BWM, emerging economy context, and barriers to IS adoption are amalgamated in a singular research framework. This proposed method not only ranks the barriers but also captures their relative importance, allowing for a more nuanced understanding of their impact. The application of Bayesian BWM highlights the significance of considering both the best and the worst criteria, which is crucial for robust decision-making. Incorporating expert judgments through Bayesian analysis enhances the validity and reliability of the findings. This approach ensures that the identified barriers are grounded in the collective expertise of apparel industry professionals, making the results more actionable for industry practitioners, policymakers, and researchers.

### 5.2 Practical implications

In response to the implementation challenges of IS in Bangladesh's AMS, this study recommends a comprehensive, policy-focused approach. Recognizing the sector's pivotal role in Bangladesh's economy, the study suggests that IS essential to balancing economic growth with environmental sustainability. Strategic actions are necessary to overcome barriers such as *lack of technology and infrastructure readiness*, *lack of inter-company cooperation,* and *lack of management* support. The study advises policymakers to focus on initiatives that enhance technological capabilities and infrastructure, which is critical for efficient IS. It recommends creating platforms for collaboration and knowledge exchange within the industry, which is essential for fostering partnerships and sharing best practices. Additionally, the study emphasizes the importance of educational programs and workshops to develop IS expertise among AMS stakeholders.

Promoting inter-company cooperation through incentives and regulatory frameworks is crucial for developing symbiotic relationships. The study also stresses the need for robust monitoring systems and continuous feedback mechanisms to track and improve IS practices. Moreover, it highlights the importance of management support in creating an environment conducive to sustainable practices. Industry leaders are encouraged to invest in technological innovation, build partnerships, and commit to management advocacy to enhance AMS's resilience, resource efficiency, and environmental performance.

By adopting IS and circular economy principles, the sector can position itself at the forefront of sustainable development, appealing to eco-conscious consumers and gaining a competitive edge globally. The study provides actionable insights for shaping strategies that address these barriers, enabling stakeholders to utilize IS for sustainable industrial growth and contributing to circular economy practices and environmental preservation. Adapting successful global IS models to the local context offers additional valuable insights. This comprehensive strategy, encompassing policy development, collaboration, education, monitoring, leadership, and global best practices, offers a detailed guide for policymakers and industry leaders to navigate IS implementation challenges, promoting sustainable industrial growth and environmental conservation in emerging economies like Bangladesh.

### 5.3 Implications for sustainability

IS, as a collaborative approach among industries, holds great potential for enhancing resource efficiency, curbing waste generation, and safeguarding the environment. Particularly in a context where economic growth often occurs at the expense of environmental considerations, incorporating IS within the AMS presents an avenue for achieving a harmonious equilibrium between economic advancement and sustainable practices. By effectively implementing IS strategies, it becomes feasible to contribute significantly to the materialization of various significant SDGs. For example, addressing barriers like *lack of technology and infrastructure readiness* and *economic and technological infeasibility* will help to achieve SDG 8 (Decent Work and Economic Growth). Again, addressing barriers like *lack of inter-company cooperation* and *lack of management* support will help in the achievement of SDG 9 (Promote Inclusive and Sustainable Industrialization).

By mitigating these barriers, strides can be made toward realizing the goal of inclusive and sustainable industrialization. Sustainable consumption and production patterns (SDG 12) can also be advanced through targeted efforts such as minimizing *high processing costs* and effectively managing the *disruption of availability*. Again, SDG 13 (Take Urgent Action to Combat Climate Change) benefits from resolving barriers such as *lack of legal requirements* and *ecological safety barriers*. Lastly, SDG 15 (Sustainable Use of Land) can be attained by addressing the barrier associated with *waste disposal costs*, a crucial step towards ensuring responsible land use.

## 6 Conclusions

Over the past few decades, IS have garnered significant attention for their potential to drive sustainable industrial practices by improving resource efficiency, minimizing waste, and safeguarding the environment through collaborative exchanges among industries. This paradigm shift has taken on particular relevance in emerging economies like Bangladesh, where integrating sustainable practices into the AMS promises to strike a delicate balance between economic growth and environmental sustainability. This sector contributes the lion's share to the economic growth of Bangladesh and, at the same time, generates substantial waste and pollution output that adversely affects the environment and local communities. Thus, the successful implementation of IS practices is crucial. However, obstacles to this integration make execution more complicated.

Therefore, this study intended to shed light on the critical barriers hindering the integration of IS practices in the AMS. Through a structured framework developed by drawing insights from extensive literature reviews and professional interviews, 17 distinct barriers were systematically identified and categorized into four overarching groups. Then, the Bayesian BWM approach was utilized to prioritize them. Our findings reveal that *“lack of technology and infrastructure readiness”* is the most pressing barrier. This underscores the urgent need for investments and advancements in technology and infrastructure to enable effective symbiotic exchanges within the sector. Barriers like *“lack of inter-company cooperation”* and *“lack of management* support*”* also emerged as crucial, emphasizing the importance of cultivating collaborative networks with support from the management and stakeholders.

The implications of the study's findings are profound and multifaceted. This study takes a step forward by incorporating both the Bayes theorem and the sophisticated BWM approach, thus presenting a more contemporary framework. To the best of our knowledge, this represents a pioneering instance wherein Bayesian BWM, emerging economy context, and barriers to IS adoption are amalgamated in a singular research framework. Policymakers should prioritize technological advancements, incentivize inter-company cooperation, and foster management support to promote sustainability. Industry leaders and stakeholders should invest in technology, build partnerships, and advocate for management commitment. These steps boost resilience, resource efficiency, and environmental performance, positioning them as sustainability leaders. The collaborative approach of IS has the potential to significantly contribute to the realization of key Sustainable Development Goals (SDGs) such as economic growth, sustainable industrialization, responsible consumption and production, climate action, and sustainable land use.

This study also has some drawbacks, which can be addressed in future research attempts. The study especially emphasizes the context of an emerging economy, which may limit the findings' potential for universal application. Hence, other economic perspectives should be explored in the future as well. Again, the data used to construct the presented approach came mostly from apparel industry professionals. However, it's probable that a few biases in judgment probably seeped into the results, which might not precisely reflect reality. Again, this study only considered 17 significant barriers. More new significant barriers may arise in the future, and this research work can be extended further considering the updated barriers. Moreover, in the future, the factors can be categorized using factor analysis for improved accuracy.

Our research methodology was crafted to effectively gather a broad spectrum of opinions within the AMS in Bangladesh. However, it's important to acknowledge that it might not capture every possible perspective. The criteria for selecting experts and data were primarily based on their availability and relevance to our area of expertise, which might lead to selection bias. Moreover, while our sample size was deemed sufficient for conducting Bayesian BWM analysis, it may not fully reflect the AMS's diverse landscape, possibly affecting the universality of our study's applicability.

The Bayesian BWM is recognized for its capacity to navigate the complexities of decision-making by amalgamating expert opinions with probabilistic analysis. Nonetheless, the efficacy of this method is contingent upon the reliability and objectivity of the expert feedback, which is inherently variable and could introduce a level of subjectivity. The Bayesian technique's strength in handling uncertainty also comes with the prerequisite of presuming certain prior distributions, potentially influencing the results. Additionally, the intricacies of Bayesian BWM demand a specific proficiency in Bayesian statistics for accurate interpretation and validation of findings.

Future studies, therefore, should attempt to expand the sampling strategy to encompass a wider and more varied set of opinions within the AMS in Bangladesh, improving the research's representativeness and applicability. Implementing randomized expert selection methods could mitigate selection bias, offering a more equitable overview of the obstacles to adopting IS. Again, examining different prior distributions and incorporating an expanded set of expert opinions might enhance the Bayesian BWM model's precision and diminish subjectivity. Finally, in the future, researchers can validate the construct proposed in this study using statistical validation techniques such as PLS-SEM (Partial least squares with structural equation modeling) to enhance the robustness of the study.

## Data availability

All data used in this research are provided in the article and in the supplementary materials file.

## CRediT authorship contribution statement

**Mosaddeque Hossain:** Writing – original draft, Software, Methodology, Investigation, Formal analysis, Data curation, Conceptualization. **Ridwan Al Aziz:** Writing – review & editing, Visualization, Validation, Supervision, Resources. **Chitra Lekha Karmaker:** Writing – review & editing, Writing – original draft, Visualization, Software, Methodology, Investigation, Formal analysis. **Binoy Debnath:** Writing – review & editing, Visualization, Methodology, Investigation. **A.B. M. Mainul Bari:** Writing – review & editing, Visualization, Validation, Supervision, Resources. **Abu Reza Md Towfiqul Islam:** Writing – review & editing.

## Declaration of competing interest

The authors declare that they have no known competing financial interests or personal relationships that could have appeared to influence the work reported in this paper.
