# Supplementary material for: Exploring the barriers to implement industrial symbiosis in the apparel manufacturing industry: Implications for sustainable development
Source: Heliyon. 2024 Jul 5;10(13):e34156. doi: 10.1016/j.heliyon.2024.e34156 (PMC11277746; doi:10.1016/j.heliyon.2024.e34156)
Supplement: Multimedia component 1 [file mmc1.docx]

**Supplementary Materials**

**Appendix A: Questionnaire for Determining the Related Key Barriers to Implementing Industrial Symbiosis (IS) in the Apparel Manufacturing Sector.**

***Q.1:*** *What role do you represent in the apparel manufacturing sector?*

***Q.2:*** *Mention the years of experience you have in the apparel manufacturing sector.*

***Q.3:*** *Please select the key barriers to implementing industrial symbiosis (IS) in the apparel manufacturing sector from the list compiled below. If a barrier does have a substantial impact, please choose "Yes"; otherwise, select "No". Additionally, you are welcome to provide any additional barriers that you think are essential to implementing IS in Bangladesh's apparel manufacturing sector.*

| **SL** | **Key Barriers** | **Put "Yes" if relevant and "No" if irrelevant** |
| --- | --- | --- |
| 1. | High processing cost |  |
| 2. | High logistics cost |  |
| 3. | Excessive Supply |  |
| 4. | Shortage of Supply |  |
| 5. | Lack of funding to promote IS |  |
| 6. | Market immaturity |  |
| 7. | Lack of management support |  |
| 8. | Lack of inter-company cooperation |  |
| 9. | Lack of trust among the locators |  |
| 10. | Personal barriers to initiating IS |  |
| 11. | Lack of research and groundwork |  |
| 12. | Lack of awareness of the IS concept |  |
| 13. | Lack of technology and infrastructure readiness |  |
| 14. | Economic and technological unfeasibility |  |
| 15. | Lack of legal requirements |  |
| 16. | Lack of policy to incentivize IS |  |
| 17. | Ecological Safety barrier |  |
| 18. | Low waste disposal cost |  |

**Appendix B: Best to Others Vectors**

**Table B1: '**Best to Others' vector for the clusters of barriers.

| ***Experts*** | ***Most significant barrier*** | ***C1*** | ***C2*** | ***C3*** | ***C4*** |
| --- | --- | --- | --- | --- | --- |
| Expert 1 | C3. Cognitive and Technological barriers | 9 | 3 | 1 | 6 |
| Expert 2 | C2. Management related barriers | 3 | 1 | 5 | 9 |
| Expert 3 | C1. Economic barriers | 1 | 9 | 3 | 5 |
| Expert 4 | C3. Cognitive and Technological barriers | 9 | 7 | 1 | 4 |
| Expert 5 | C3. Cognitive and Technological barriers | 4 | 3 | 1 | 9 |
| Expert 6 | C3. Cognitive and Technological barriers | 4 | 5 | 1 | 9 |
| Expert 7 | C1. Economic barriers | 1 | 9 | 3 | 4 |
| Expert 8 | C3. Cognitive and Technological barriers | 9 | 4 | 1 | 3 |
| Expert 9 | C2. Management related barriers | 3 | 1 | 6 | 9 |
| Expert 10 | C1. Economic barriers | 1 | 3 | 9 | 7 |
| Expert 11 | C3. Cognitive and Technological barriers | 4 | 3 | 1 | 9 |
| Expert 12 | C2. Management related barriers | 3 | 1 | 5 | 9 |

**Table B2: '**Best to Others' vector for the *Economic barriers*.

| ***Experts*** | ***Most significant barrier*** | ***EB1*** | ***EB2*** | ***EB3*** | ***EB4*** | ***EB5*** |
| --- | --- | --- | --- | --- | --- | --- |
| Expert 1 | EB2. High logistic cost | 1 | 2 | 9 | 6 | 4 |
| Expert 2 | EB2. High logistics cost | 3 | 1 | 9 | 5 | 6 |
| Expert 3 | EB4. Lack of funding to promote IS | 9 | 4 | 5 | 1 | 3 |
| Expert 4 | EB4. Lack of funding to promote IS | 6 | 9 | 2 | 1 | 7 |
| Expert 5 | EB1. High processing cost | 1 | 7 | 8 | 4 | 9 |
| Expert 6 | EB3. Disruption of availability | 3 | 4 | 1 | 6 | 9 |
| Expert 7 | EB1. High processing cost | 1 | 3 | 9 | 5 | 4 |
| Expert 8 | EB5. Market immaturity | 3 | 4 | 6 | 9 | 1 |
| Expert 9 | EB1. High processing cost | 1 | 2 | 9 | 8 | 4 |
| Expert 10 | EB2. High logistics cost | 2 | 1 | 8 | 9 | 5 |
| Expert 11 | EB1. High processing cost | 1 | 2 | 7 | 9 | 6 |
| Expert 12 | EB1. High processing cost | 1 | 3 | 9 | 7 | 5 |

**Table B3: '**Best to Others' vector for the *Management-related barriers*.

| ***Experts*** | ***Most significant barrier*** | ***MB1*** | ***MB2*** | ***MB3*** | ***MB4*** |
| --- | --- | --- | --- | --- | --- |
| Expert 1 | MB2. Lack of inter-company cooperation | 3 | 1 | 4 | 9 |
| Expert 2 | MB2. Lack of inter-company cooperation | 3 | 1 | 9 | 5 |
| Expert 3 | MB1. Lack of management support | 1 | 3 | 6 | 9 |
| Expert 4 | MB3. Lack of trust among the locators | 9 | 3 | 1 | 4 |
| Expert 5 | MB1. Lack of management support | 1 | 5 | 3 | 9 |
| Expert 6 | MB2. Lack of inter-company cooperation | 4 | 1 | 3 | 9 |
| Expert 7 | MB1. Lack of management support | 1 | 3 | 5 | 9 |
| Expert 8 | MB1. Lack of management support | 1 | 5 | 9 | 3 |
| Expert 9 | MB2. Lack of inter-company cooperation | 2 | 1 | 6 | 9 |
| Expert 10 | MB1. Lack of management support | 1 | 3 | 7 | 9 |
| Expert 11 | MB2. Lack of inter-company cooperation | 2 | 1 | 9 | 7 |
| Expert 12 | MB2. Lack of inter-company cooperation | 3 | 1 | 9 | 6 |

**Table B4: '**Best to Others' vector for the *Cognitive and Technological barriers*.

| ***Experts*** | ***Most significant barrier*** | ***CT1*** | ***CT2*** | ***CT3*** | ***CT4*** |
| --- | --- | --- | --- | --- | --- |
| Expert 1 | CT1. Lack of research and groundwork | 1 | 9 | 3 | 5 |
| Expert 2 | CT1. Lack of research and groundwork | 1 | 9 | 5 | 4 |
| Expert 3 | CT2. Lack of awareness of IS concept | 4 | 1 | 9 | 7 |
| Expert 4 | CT1. Lack of research and groundwork | 1 | 3 | 4 | 9 |
| Expert 5 | CT3. Lack of technology and infrastructure readiness | 3 | 5 | 1 | 9 |
| Expert 6 | CT3. Lack of technology and infrastructure readiness | 6 | 9 | 1 | 3 |
| Expert 7 | CT4. Economic and technological unfeasibility | 7 | 9 | 2 | 1 |
| Expert 8 | CT1. Lack of research and groundwork | 1 | 7 | 9 | 4 |
| Expert 9 | CT3. Lack of technology and infrastructure readiness | 6 | 9 | 1 | 3 |
| Expert 10 | CT4. Economic and technological unfeasibility | 6 | 9 | 2 | 1 |
| Expert 11 | CT3. Lack of technology and infrastructure readiness | 7 | 9 | 1 | 3 |
| Expert 12 | CT3. Lack of technology and infrastructure readiness | 3 | 9 | 1 | 4 |

**Table B5: '**Best to Others' vector for the *Environmental and Policy barriers*.

| ***Experts*** | ***Most significant barrier*** | ***EP1*** | ***EP2*** | ***EP3*** | ***EP4*** |
| --- | --- | --- | --- | --- | --- |
| Expert 1 | EP2. Lack of policy to incentivize IS | 9 | 1 | 5 | 3 |
| Expert 2 | EP2. Lack of policy to incentivize IS | 2 | 1 | 4 | 9 |
| Expert 3 | EP2. Lack of policy to incentivize IS | 3 | 1 | 5 | 9 |
| Expert 4 | EP4. Low waste disposal cost | 9 | 3 | 4 | 1 |
| Expert 5 | EP4. Low waste disposal cost | 4 | 4 | 9 | 1 |
| Expert 6 | EP1. Lack of legal requirements | 1 | 3 | 9 | 4 |
| Expert 7 | EP2. Lack of policy to incentivize IS | 3 | 1 | 5 | 9 |
| Expert 8 | EP2. Lack of policy to incentivize IS | 4 | 1 | 2 | 9 |
| Expert 9 | EP2. Lack of policy to incentivize IS | 6 | 1 | 9 | 3 |
| Expert 10 | EP4. Low waste disposal cost | 7 | 3 | 9 | 1 |
| Expert 11 | EP2. Lack of policy to incentivize IS | 9 | 1 | 6 | 3 |
| Expert 12 | EP1. Lack of legal requirements | 1 | 3 | 6 | 9 |

**Appendix C: Others to Worst Vectors**

**Table C1: '**Others to Worst' vector for the clusters of barriers.

| ***Experts*** | ***Least significant barrier*** | ***C1*** | ***C2*** | ***C3*** | ***C4*** |
| --- | --- | --- | --- | --- | --- |
| Expert 1 | C1. Economic barriers | 1 | 4 | 9 | 2 |
| Expert 2 | C4. Environmental and Policy barriers | 6 | 9 | 3 | 1 |
| Expert 3 | C2. Management related barriers | 9 | 1 | 6 | 4 |
| Expert 4 | C2. Management related barriers | 3 | 1 | 9 | 8 |
| Expert 5 | C4. Environmental and Policy barriers | 7 | 8 | 9 | 1 |
| Expert 6 | C4. Environmental and Policy barriers | 7 | 6 | 9 | 1 |
| Expert 7 | C2. Management related barriers | 9 | 1 | 6 | 4 |
| Expert 8 | C1. Economic barriers | 1 | 6 | 9 | 7 |
| Expert 9 | C4. Environmental and Policy barriers | 8 | 9 | 5 | 1 |
| Expert 10 | C3. Cognitive and Technological barriers | 9 | 7 | 1 | 3 |
| Expert 11 | C4. Environmental and Policy barriers | 7 | 5 | 9 | 1 |
| Expert 12 | C4. Environmental and Policy barriers | 6 | 9 | 4 | 1 |

**Table C2: '**Others to Worst' vector for the *Economic barriers.*

| ***Experts*** | ***Least significant barrier*** | ***EB1*** | ***EB2*** | ***EB3*** | ***EB4*** | ***EB5*** |
| --- | --- | --- | --- | --- | --- | --- |
| Expert 1 | EB3. Disruption of Availability | 9 | 7 | 1 | 3 | 6 |
| Expert 2 | EB3. Disruption of availability | 7 | 9 | 1 | 6 | 3 |
| Expert 3 | EB1. High processing cost | 1 | 3 | 5 | 9 | 7 |
| Expert 4 | EB2. High logistics cost | 5 | 1 | 7 | 9 | 2 |
| Expert 5 | EB5. Market immaturity | 9 | 5 | 2 | 7 | 1 |
| Expert 6 | EB5. Market immaturity | 6 | 4 | 9 | 3 | 1 |
| Expert 7 | EB3. Disruption of availability | 9 | 8 | 1 | 4 | 6 |
| Expert 8 | EB4. Lack of funding to promote IS | 8 | 7 | 4 | 1 | 9 |
| Expert 9 | EB3. Disruption of availability | 9 | 7 | 1 | 3 | 5 |
| Expert 10 | EB4. Lack of funding to promote IS | 8 | 9 | 3 | 1 | 5 |
| Expert 11 | EB4. Lack of funding to promote IS | 9 | 8 | 3 | 1 | 4 |
| Expert 12 | EB3. Disruption of availability | 9 | 8 | 1 | 3 | 6 |

**Table C3: '**Others to Worst' vector for the *Management Related barriers.*

| ***Experts*** | ***Least significant barrier*** | ***MB1*** | ***MB2*** | ***MB3*** | ***MB4*** |
| --- | --- | --- | --- | --- | --- |
| Expert 1 | MB4. Personal barrier | 7 | 9 | 4 | 1 |
| Expert 2 | MB3. Lack of trust among the locators | 7 | 9 | 1 | 3 |
| Expert 3 | MB4. Personal barrier | 9 | 6 | 4 | 1 |
| Expert 4 | MB1. Lack of management support | 1 | 5 | 9 | 6 |
| Expert 5 | MB4. Personal barrier | 9 | 6 | 7 | 1 |
| Expert 6 | MB4. Personal barrier | 4 | 9 | 6 | 1 |
| Expert 7 | MB4. Personal barrier | 9 | 4 | 2 | 1 |
| Expert 8 | MB3. Lack of trust among the locators | 9 | 6 | 1 | 8 |
| Expert 9 | MB3. Lack of trust among the locators | 7 | 9 | 1 | 2 |
| Expert 10 | MB4. Personal barrier | 9 | 7 | 2 | 1 |
| Expert 11 | MB3. Lack of trust among the locators | 7 | 9 | 1 | 3 |
| Expert 12 | MB3. Lack of trust among the locators | 6 | 9 | 1 | 3 |

**Table C4: '**Others to Worst' vector for the *Cognitive and Technological barriers.*

| ***Experts*** | ***Least significant barrier*** | ***CT1*** | ***CT2*** | ***CT3*** | ***CT4*** |
| --- | --- | --- | --- | --- | --- |
| Expert 1 | CT2. Lack of awareness of IS concept | 9 | 1 | 6 | 4 |
| Expert 2 | CT2. Lack of awareness of IS concept | 9 | 1 | 5 | 7 |
| Expert 3 | CT3. Lack of technology and infrastructure readiness | 6 | 9 | 1 | 2 |
| Expert 4 | CT3. Economic and technological unfeasibility | 9 | 7 | 6 | 1 |
| Expert 5 | CT3. Economic and technological unfeasibility | 7 | 5 | 9 | 1 |
| Expert 6 | CT2. Lack of awareness of IS concept | 4 | 1 | 9 | 7 |
| Expert 7 | CT2. Lack of awareness of IS concept | 2 | 1 | 8 | 9 |
| Expert 8 | CT3. Lack of technology and infrastructure readiness | 9 | 5 | 1 | 6 |
| Expert 9 | CT2. Lack of awareness of IS concept | 4 | 1 | 9 | 6 |
| Expert 10 | CT2. Lack of awareness of IS concept | 3 | 1 | 7 | 9 |
| Expert 11 | CT2. Lack of awareness of IS concept | 4 | 1 | 9 | 7 |
| Expert 12 | CT2. Lack of awareness of IS concept | 5 | 1 | 9 | 2 |

**Table C5: '**Others to Worst' vector for the *Environmental and Policy barriers.*

| ***Experts*** | ***Least significant barrier*** | ***EP1*** | ***EP2*** | ***EP3*** | ***EP4*** |
| --- | --- | --- | --- | --- | --- |
| Expert 1 | EP1. Lack of legal requirements | 1 | 9 | 4 | 6 |
| Expert 2 | EP4. Low waste disposal cost | 6 | 9 | 5 | 1 |
| Expert 3 | EP4. Low waste disposal cost | 6 | 9 | 3 | 1 |
| Expert 4 | EP1. Lack of legal requirements | 1 | 7 | 6 | 9 |
| Expert 5 | EP3. Ecological Safety barrier | 6 | 6 | 1 | 9 |
| Expert 6 | EP3. Ecological Safety barrier | 9 | 5 | 1 | 2 |
| Expert 7 | EP4. Low waste disposal cost | 4 | 9 | 2 | 1 |
| Expert 8 | EP4. Low waste disposal cost | 6 | 9 | 7 | 1 |
| Expert 9 | EP3. Ecological Safety barrier | 3 | 9 | 1 | 6 |
| Expert 10 | EP3. Ecological Safety barrier | 4 | 7 | 1 | 9 |
| Expert 11 | EP1. Lack of legal requirements | 1 | 9 | 4 | 7 |
| Expert 12 | EP4. Low waste disposal cost | 9 | 6 | 3 | 1 |
